# Patients’, families’ and healthcare providers’ perspectives on end-of-life communication in Chinese hospital settings: A qualitative study protocol

**DOI:** 10.1371/journal.pone.0296342

**Published:** 2023-12-27

**Authors:** Weilin Chen, Joyce Oi Kwan Chung, Katherine Ka Wai Lam, Alex Molassiotis

**Affiliations:** 1 School of Nursing, The Hong Kong Polytechnic University, Kowloon, Hong Kong Special Administrative Region, China; 2 Health and Social Care Research Centre, University of Derby, Derby, United Kingdom; University of Toronto Temerty Faculty of Medicine, CANADA

## Abstract

**Background:**

Perspectives of key stakeholders should be fully considered to enhance culturally appropriate strategies in end-of-life communication and strengthen healthcare service delivery. So far, little research evidence is available on Chinese patients’, families’, and healthcare professionals’ experiences with and perspectives of end-of-life communication in hospital settings.

**Aim:**

The current study aims to explore experiences, perceptions and suggestions of end-of-life communication among Chinese terminally ill patients, their families and healthcare providers.

**Methods:**

The phenomenology qualitative approach will be adopted. Semi-structured in-depth interviews and focus group discussions will be used to collect relevant data. Eligible terminally ill patients, family caregivers and healthcare providers will be recruited in two hospitals in Mainland China via purposive sampling. Thematic analysis will be performed to analyse data. The Standards for Reporting Qualitative Research (SRQR) checklist will be followed for reporting. This study has been registered at ClinicalTrials.gov (NCT05734781).

**Discussion:**

This qualitative study is, as far as we are aware, the first to specifically address patient/family-provider end-of-life communication in the Chinese social-cultural context. The results hold the potential to enrich current knowledge of end-of-life communication, navigate culturally appropriate communication strategies, and inform the development of related training programs for healthcare providers in hospital settings.

## Introduction

It has been found in a systematic review included 20 American studies that effective end-of-life communication is associated with quality end-of-life care, such as lower end-of-life care costs (median US$1,048 vs US$23,482; *P* < .001); less intensive care near end-of-life (ORs 0.26–0.68); and more hospice use (ORs 1.79–6.88) [1]. End-of-life communication refers to a series of highly individualised conversations regarding death and dying, such as disclosure of prognosis, advance care planning, and goals of care discussions [2–5]. It is an ongoing and iterative process and is also related to a patient’s transition to different settings [6].

Early end-of-life communication is widely advocated in the context of a serious illness, meaning such conversations should be started as soon as a patient has been identified as dying [1, 6–9]. Hospitals are common institutions where some patients are diagnosed with end-of-life conditions, seek any possible treatments and transfer to other healthcare settings [6, 10]. Given this, healthcare providers in hospital settings play an essential role in initiating and facilitating end-of-life communication [11, 12]. They are expected to accommodate patients’ preferences for information and shared decision-making while being sensitive to their emotional needs and vulnerabilities [13]. However, partly due to the curative culture and acquiescence of life-sustaining treatments, end-of-life communication has often been neglected or occurs very close to patients’ deaths in hospital settings [6, 9].

As the significance and benefits of end-of-life communication have been recognised in recent years, it is suggested to be integrated into routine clinical procedures for patients with serious illnesses [9]. Understanding the accounts of main stakeholders directly involved in end-of-life communication is a priority to organise meaningful communicative practice in a systematic approach and embed it in practice regularly [14]. Notably, existing research has primarily investigated from the vantage point of healthcare professionals, especially physicians [15–18]. There is a shortage of evidence about end-of-life communication from the views of other stakeholders, particularly patients with advanced diseases and relatives of such patients. Parker et al. [19] reviewed patients’ and their families’ information preferences regarding dying, life expectancy and future care. They discovered that the evidence is largely made up of studies conducted in specific populations from English-speaking and Northern European countries which may not be generalisable to people from different cultural backgrounds. O’Connor et al. [20] explored hospitalised patients’ experiences of goals of care discussion and decision-making process, which is only a part of end-of-life communication. Patient and family experiences and perspectives on the end-of-life communication process need further exploration and analysis to support the development of effective end-of-life communication system, enhance education and training for healthcare professionals in hospital settings, and foster the improvement of communication practice.

Our previous review of the international literature on end-of-life communication strategies emphasised the imbalance between the amount of evidence from Anglo-Saxon and non-Anglo-Saxon nations, which hinders a thorough comprehension and comparison of end-of-life communication in various sociocultural situations [21]. Moreover, recent research findings reinforce the influence of Chinese culture and philosophy on end-of-life communication. For instance, the traditional understandings and conceptualisations of death (e.g., discussion about dying and death is taboo and regarded as unlucky and harmful) impede the promotion of end-of-life communication; and the family-centred decision-making style may conflict with patient’s autonomy [15]. Previous studies explored the working experiences of Chinese physicians and nurses who work with advanced cancer patients [22, 23]; nurses’ attitudes towards death and dying [24] and end-of-life care [25]; family communication experiences [26]; patients’ understanding of death [27] and advance directive preferences [28]. Nevertheless, little is known about Chinese patients, families and healthcare providers’ experiences and perceptions of client-provider end-of-life communication. In addition, no empirical evidence on end-of-life communication strategies from a Chinese perspective has been found.

## Materials and methods

### Aims and objectives

This study aims to explore stakeholders’ (patients, families and healthcare providers) perspectives on end-of-life communication in Chinese hospital settings. More specific objectives are to (1) describe end-of-life communication experiences of Chinese terminally ill patients, families and healthcare providers, (2) understand their perceptions, attitudes and beliefs of end-of-life communication, (3) collect their suggestions and expectations on improving end-of-life communication, and (4) develop culturally appropriate communication strategies in the Chinese social-cultural context. The research questions are as follows: (1) what experiences do Chinese terminally ill patients, families and healthcare providers have in end-of-life communication in hospital settings? (2) what are their perceptions, attitudes and beliefs of end-of-life communication? (3) what are their suggestions and expectations for improving end-of-life communication? (4) what are the culturally appropriate communication strategies in the Chinese social-cultural context?

### Study design and setting

A phenomenology qualitative approach will be performed, which emphasizes deriving meaning from comprehensive descriptions of lived experiences related to a phenomenon [29]. We will adopt face-to-face interviews and focus group discussions to unearth participants’ experiences and perspectives. Semi-structured in-depth interviews with patients and family caregivers will be conducted respectively. In-depth interviews are helpful when the study is designed to understand and generate in-depth personal accounts [30]. Due to sensitive end-of-life-related topics in the interviews, these interviews will be conducted separately to encourage the engagement of participants. Focus group discussions will be arranged with healthcare providers, allowing them to discuss end-of-life communication and generate group interactions and creative thinking [30]. To avoid power differentials and professionally based hierarchies that may affect participation negatively [31], focus group discussions will be conducted with physicians, nurses and other healthcare professionals (such as anaesthetists and psychotherapists), respectively. For healthcare providers unwilling or unable to join the focus group discussions, semi-structured in-depth interviews will be conducted. Eligible patients, family caregivers and healthcare providers will be recruited in two hospitals in Hangzhou, China. The two hospitals are large public Grade-A tertiary hospitals (the highest level of hospitals in mainland China) and receive patients from all over the country. The departments involved will be oncology, radiotherapy, cardiology, hematology, nephrology, respiratory and critical care medicine.

### Participants

Purposive sampling methods will be adopted. The variation of participants’ characteristics (such as gender, age and education level of patients and families; disciplines of healthcare providers) will be considered as much as possible to ensure the breadth of relevant views [32]. Besides, both cancer and non-cancer patients will be included as they are both key informants with the knowledge or experience of our interest phenomenon [3, 33]. There is no gold standard on how large the sample size would be adequate in qualitative research. Thus, the sample size in this study will be decided following the principle of information saturation, which means the recruitment of participants will stop when the research topic has been thoroughly investigated [34]. Generally, consider 5 to 25 participants to provide sufficient data [35]. For a medium-sized research study, three to six focus groups are deemed adequate [30]. Around 20 patients and 20 family caregivers will be interviewed, and 3 to 6 focus groups, 4 to 8 participants each, will be arranged for reference in this study.

Included patients should: (1) Be aged older than 18 years. (2) Have a defined incurable life-limiting illness with a likely life expectancy of fewer than 12 months [36], according to the consultation with the patient’s physician. (3) Have been informed of disease diagnosis and treatment. (4) Be able to speak Mandarin and express clearly. (5) Be able to provide informed consent. Included family caregivers should: (1) Be aged older than 18 years. (2) One of the terminally ill patient’s primary family caregivers who are familiar with the patient’s situation, which is defined in this study as lived with or visiting patients at least twice a week in the past month. (3) Have been informed of the patient’s disease diagnosis and treatment. (4) Be able to speak Mandarin. (5) Be able to provide informed consent. Patients and family caregivers with severe auditory or cognitive impairment will be excluded. Included healthcare providers should: (1) Be nurses, physicians or other healthcare professionals. (2) Be experienced in providing treatment or care to patients with advanced, life-limiting illnesses (whose likely life expectancies of fewer than 12 months) and family caregivers of such patients for at least five years [37]. (3) Consent to participate in the study.

When approaching the eligible participants, firstly, the researcher will contact the relevant department head for consent and permission to enter the ward. Secondly, patients, family caregivers, and healthcare providers who meet the inclusion and exclusion criteria will be approached and recruited by the first author. Before approaching patients, the patient’s physician will be consulted on patient eligibility (patient’s medical record will be accessed by the physician and reviewed together by the first author, if necessary) and be sought approval on the patient’s possible participation in interviews. Additionally, a promotional poster detailing the nature and purpose of the research project will be posted on the Hospital’s notice board to attract possible participants who can self-select to partake in and contact researchers. Each eligible informant will be met in person to foster relationship development, build trust, enable inquiries and clarification requests.

### Data collection procedures

The recruitment was started on May 4, 2023, and the data collection is expected to complete in October 2023. Participants will have opportunities to select a time and location which is suitable for them. Meanwhile, a quiet and private interview room will be offered. Focus group discussions will be carried out in a booked conference room. The interviews and discussions will be facilitated by the first author, a female Chinese registered nurse (RN) and PhD candidate who had conducted qualitative studies and has received qualitative interviewing training from supervisors before data collection. The interview guides (Table 1) for interviews and focus group discussions are developed by the research team based on literature related to end-of-life communication [2, 3, 5, 6, 37–42]. The interview questions are designed in line with the research objectives and intended to invite various stakeholders to share end-of-life communication experiences, perceptions, strategies and suggestions in an open and supportive manner. The researcher will follow the interview and discussion process using prompts, including four main sub-topics of end-of-life communication, i.e., diagnosis and prognosis; advance care planning; goals of care; and death and dying [2, 3, 5, 6, 38], to ensure meaningful interviews and discussions. One or two patient-, family caregiver- pilot interviews and one pilot focus group discussion will be performed to troubleshoot issues with the interview process and questions and revise the interview guides if necessary [26, 43]. Pilot interviews will also aid in skilful and effective questioning in the formal study [44]. Sociodemographic data about participants will be collected via a brief self-developed questionnaire (S1 Appendix) at the end of each interview or focus group discussion. Each interview and focus group discussion will be expected to last 30–60 and 60–90 minutes, respectively. The interviews and discussions will be audio-recorded and transcribed verbatim in Mandarin first during data collection.

**Table 1:** Interview guides.

Patients and families	
Questions	Prompts
Experience	
Tell me about any communication experiences that you have had with healthcare providers.	What topics were covered?How did you communicate…? • Diagnosis and prognosis *(e*.*g*., *health condition; clinical course; treatment uncertainties and limitations; and life expectancy)* • Advance care planning *(i*.*e*., *discussions of future care; may include the individual’s concerns*, *wishes*, *values*, *understandings*, *and preferences)* • Goals of care *(i*.*e*., *discussions of current care and decisions; may include specific medical interventions*, *such as whether to utilise life-sustaining treatments)* • Death and dying *(e*.*g*., *end-of-life wishes; care for psychological*, *spiritual*, *and existential problems; arrangements after patients’ death; and bereavement support)* Who was present?When and where did the communication occur?
How did you feel about the communication?	Is anything impressive or annoying during the communication process? Why?
What do you think of the communication?	Are they good? Why?
Perception, attitude and belief	
If your (the patient’s) condition continues getting worse, or the curative treatment effect is not satisfactory, do you think it is necessary for healthcare providers to have end-of-life communication with you?	Why or why not?
What is your understanding of end-of-life communication?	Is anything important for you to discuss with healthcare providers?
Strategy	
How would you like the healthcare provider to have end-of-life communication with you?	How to communicate…? • Diagnosis and prognosis • Advance care planning • Goals of care • Death and dying What specific words or phrases do you think healthcare providers should use? Would you like to give me an example? Who do you think should initiate end-of-life communication with you? Who should be involved? When and where are appropriate?
Suggestion	
If you are invited to give suggestions to healthcare providers who would like to have end-of-life communication with you, what would you say?	E.g., for healthcare providers who do not have much experience in end-of-life communication
**Healthcare providers**	
Questions	Prompts
Perception, attitude and belief	
What is your understanding of end-of-life communication?	What topics include? E.g., disclosure of diagnosis and prognosis; advance care planning; goals of care discussions; talking about death and dying, etc.
What do you think about end-of-life communication between healthcare providers and patients and their families in hospitals?	If the patient’s condition continues getting worse, or the curative treatment effect is not satisfactory, do you think it is necessary for healthcare providers to have end-of-life communication with patients and their families? Why or why not?
Experience	
Tell me about any end-of-life communication experiences in your usual work.	What topics were covered? How did you communicate…? • Diagnosis and prognosis • Advance care planning • Goals of care • Death and dying Who was present? When and where did the communication occur?
How did you feel about the communication?	Is anything impressive or difficult during the communication process? Why?
How do you think your previous end-of-life communication?	Are they good? Why?
Strategy	
What, if any, specific strategies do you have that may be helpful for improving end-of-life communication between healthcare providers and patients and their families?	How to communicate…? • Diagnosis and prognosis • Advance care planning • Goals of care • Death and dying What specific words or phrases do you tend to use? Why? Would you like to give me an example? Who do you think should initiate end-of-life communication? Who should be involved? When and where are appropriate?
Suggestion	
What, if any, suggestions do you have for improving end-of-life communication between healthcare providers and patients and their families?	Are any other end-of-life issues that should be discussed? Any ideas about multi-professional end-of-life communication?
If you have the opportunity to participate in end-of-life communication skills training, what suggestions and expectations do you have?	What do you want to learn? What methods do you prefer?

### Data analysis

The data collection and analysis will be conducted simultaneously. Transcripts of interviews and focus group discussions will be entered into NVivo (QRS International, Victoria, Australia). The data analysis will be an iterative, cyclic and self-reflective process. Using inductive thematic analysis, common themes and categories will be determined through inductive reasoning and constant comparison, meaning there is no theoretical perspective to guide coding or explanation [45]. Two researchers will work independently following six interactive steps of thematic analysis: getting familiar with collected data, generating initial codes, searching for appropriate themes, reviewing these themes, defining themes, and presenting and discussing findings [45]. Any discrepancies will be discussed in a regular meeting with all research team members. A consensus will be established after discussions on potential codification differences. Participants will be given the transcriptions and analysis results for verification. The data analysis will be performed in Chinese. Two researchers fluent in Chinese and English and have experience translating qualitative research reports will translate significant statements, categories, and themes into English for reporting. To enhance accuracy, the translation results will be examined by the third researcher who is also bilingual in English and Chinese. Any disagreements in translation will be resolved through discussion. The Standards for Reporting Qualitative Research (SRQR) checklist (S2 Appendix) will be followed [46].

### Data management

All raw data will be stored securely on a password-protected computer. Paper copies of the consent form will be kept separately in a locked cabinet for five years, only accessible to the researcher team. Following the storage period, all personal information will be deleted.

Previous evidence [20, 26, 40] suggested that interviews were more helpful than detrimental and that there has not been any data to suggest that participants have suffered negative long-term consequences or have been referred for counselling due to being interviewed. Moreover, interviews may be therapeutic for participants by evoking hidden feelings and leading them to a new understanding of the events [44]. The interviews in this study will mainly include recalls of the experiences of end-of-life communication instead of real-life discussions regarding the diagnosis and prognosis of life-limiting illnesses. However, it is still likely to remind participants of past or current experiences that may elicit distress and discomfort, cause the risk of harm and precipitate emotional responses such as grief, anger, anxiety and fear. For example, patients and family caregivers may be reminded of poor prognosis or disease deterioration; emotional exhaustion or even conflict experiences in difficult communication among healthcare providers. We should consider that end-of-life communication is a sensitive topic in nature. Each people involved in sensitive research could be affected, including both participants and researchers, but avoiding it could be perceived as a failure to accept responsibility and a deprivation of participants’ agency [47]. Therefore, to minimise the risk of harm, safeguard vulnerable participants morally and ethically, and yield rich and meaningful data, some reasonable and appropriate safety measures will be taken. A risk assessment and distress protocol (Table 2) is prepared by the researchers, inspired by previous work on sensitive interviewing [44], to deal with distressed participants. Participants will be informed that they can refuse to answer questions or withdraw from the study at any time.

**Table 2:** A risk assessment and distress protocol.

Risks	Considerations	Measures
Risks to the participants		
• Patients/families/healthcare providers ask to pause or terminate the interview/discussion due to inappropriate time and/or location	Ill-suited time and/or location may make participants feel uncomfortable sharing their stories	1. Participants can withdraw from the research at any time without detriment 2. Participants will be provided with the opportunity to be interviewed at any time and location they prefer 3. Time and/or location will be rearranged if they ask. Safety, privacy and quiet will be ensured by the researcher to promote comfort
• Patients/families/healthcare providers experience distress during the interview/discussion	Discussions about end-of-life issues and related experiences may be sensitive for participants and cause distress and discomfort, which may need an immediate support	1. Participants will be asked if they would like to pause the interview, take a break and if they want to stop the audio recording. If so, such measures will be taken; necessary support will be provided, such as water and tissue; the researcher will accompany them until they are calm 2. If participants continue to show signs of upset, family or professional support (nurses, social workers and psychological consultants of the hospital) will be sought, with the participant’s consent 3. Participants decide whether to continue the interview/discussion. The interview can be rearranged for later in the day or the following day to ensure they are no longer distressed
• Patients/families/healthcare providers disclose any upsetting feelings that arise from the interview/discussion	Sensitive issues may arise from participation and have lasting effects on participants, which may need an adequate follow-up support	1. Each participant will have the opportunity to disclose to the researcher about the interview and allow for feedback after the data collection 2. Contact details of useful numbers will be offered as required. Family/peer/professional support will be advised
Risks to the researcher		
• The researcher may be at risk of emotional stress	The researcher/interviewer may experience vicarious traumatisation	1. The researcher will debrief the process with senior research team members 2. The researcher will have private time to reflect after the interview/discussion 3. Professional psychological counselling should be approached if necessary

### Quality and rigor

The credibility, dependability, confirmability and transferability of the study will be achieved by a series of strategies [48]. First, credibility will be ensured by member checking. Transcriptions and research findings will be shared with the participants to verify the accuracy of the interpretations. Second, two researchers will perform the data analysis independently. They will have comparisons and discussions of the results of their work. Any disagreements will be resolved in regular research team meetings. Third, field notes will be taken during the data collection and included in the data analysis to support interpreting the participants’ answers. A comprehensive description of the study procedure, including the study design, data collection and analysis, interpretation and report, will be included to ensure dependability. Concerning confirmability, the researchers will reflect on how their values, beliefs, and perceptions may bias the research process. The researchers will make an effort to concentrate on the participants’ stories and the meanings underlying their narratives without making any assumptions. Bracket interviews will also be completed by meeting supervisors to help the reflection process [49]. Field and self-reflective notes will be taken during the data collection and analysis to give an audit trail of context and how critical decisions on interpretation are reached [50]. To strengthen the transferability, “thick descriptions” will be adopted. An explicit study process, contextual information and direct quotes will be provided to ensure that the findings are transferable to similar contexts and populations [51].

## Discussion

To our knowledge, this qualitative study is the first to focus on patient/family-provider end-of-life communication in the Chinese context. The findings are anticipated to enrich current knowledge and understanding about end-of-life communication, specifically experiences, perceptions and suggestions of vital Chinese stakeholders. Furthermore, the study will fill the gap of no empirical evidence on end-of-life communication strategies in the Chinese context, which will be an essential foundation in refining culturally appropriate communication strategies embedded with participants’ accounts.

This study is integrated into a parent research project which aims to develop and evaluate a culturally specific end-of-life communication skills training program. The grounded data gathered in this qualitative study can be applied to support the development of the training content and enhance its cultural sensitivity, with full consideration of relevant views. The contents and effects of the training are expected to be different from previous training referring to communication curriculum [52] or model [53] derived from Western practice. In addition, the results will provide new insights into organising the end-of-life communication process in hospital settings and inform future practice and policy.

Some limitations should also be noted. First of all, it might be challenging to avoid a possible participation bias because participants who volunteer for the study are likely to be more interested in the topic and hold different opinions from those who are not interested in the topic. However, keeping the participation voluntary is essential to avoid coercion in participants’ recruitment. Also, we will include participants with as many different characteristics as possible to ensure that relevant perspectives are taken into account. Second, the included patients and family caregivers have been informed of disease diagnosis and prognosis, as we consider that it is sensitive and difficult to talk about end-of-life issues with Chinese patients and their families who have hope for a cure. This may lead us to ignore the views of end-stage patients who do not know their prognosis. Third, we used only semi-structured interviews and focus group discussions to recall participants’ past communication experiences and collecting relevant data. Such recollections and recollection-based descriptions may be inaccurate or incomplete. Other methods, such as direct participant observation, although it will be time-consuming to capture these end-of-life communications, may yield richer data.

The dissemination plan mainly includes sharing and applying research findings to practice. We will publish the results in international peer-reviewed scientific journals and present them at conferences. Key findings and practical implications will be communicated to the general public, practitioners and policymakers at domestic seminars.

## Supporting information

S1 Appendix Demographic questionnaires. (DOCX)

S2 Appendix Research checklist. (DOCX)
