# Supplementary material for: Patients’, families’ and healthcare providers’ perspectives on end-of-life communication in Chinese hospital settings: A qualitative study protocol
Source: PLoS One. 2023 Dec 27;18(12):e0296342. doi: 10.1371/journal.pone.0296342 (PMC10752523; doi:10.1371/journal.pone.0296342)
Supplement: S1 Appendix — (DOCX) [file pone.0296342.s001.docx]

**Patient characteristics**

1. Age (years)

2. Gender: Male; Female

3. Marital status: Married; Divorced/ separated; Single

4. Educational level: Primary; Lower secondary; Higher secondary; Tertiary

5. Religion: None; Buddhism; Christianity; Muslim; Other

6. Working status (before diagnosis): Employed; Unemployed

7. Diagnosis: Cancer (lung, stomach, breast, others); Non-cancer (end-stage pulmonary disease, end-stage cardiac failure, end-stage renal disease, other)

8. Treatment received: Surgery; Radiotherapy; Chemotherapy; Surgery and radiotherapy; Surgery and chemotherapy; Chemotherapy and radiotherapy; Other

9. Primary caregiver: Spouse; Parent(s); Child(ren); Other

10. Hospitalisation period (days): Less than 7 days; Equal to or longer than 7 days

11. Hospice experience: Yes; No

**Family caregiver characteristics**

1. Age (years)

2. Gender: Male; Female

3. Marital status: Married; Divorced/ separated; Single

4. Educational level: Primary; Lower secondary; Higher secondary; Tertiary

5. Religion: None; Buddhism; Christianity; Muslim; Other

6. Working status: Employed; Unemployed

7. Diagnosis of patient: Cancer (lung, stomach, breast, others); Non-cancer (end-stage pulmonary disease, end-stage cardiac failure, end-stage renal disease, other)

8. Relationship to patient: Spouse; Parent; Child; Other

**Healthcare provider characteristics**

1. Age (years)

2. Gender: Male; Female

3. Educational level: Junior college; Undergraduate; Master; Doctor

4. Religion: None; Buddhism; Christianity; Muslim; Other

5. Profession: Nurse; Physician; Other

6. Professional title: Junior; Medium; Senior

7. Department: Oncology; Geriatrics; Intensive care unit; Emergency; Outpatient

8. Working years

9. Number of years involved in the treatment or care of advanced patients
